# Differential Intracellular Protein Distribution in Cancer and Normal Cells—Beta-Catenin and CapG in Gynecologic Malignancies

**DOI:** 10.3390/cancers14194788

**Published:** 2022-09-30

**Authors:** Maria Kristha Fernandez, Molika Sinha, Malte Renz

**Affiliations:** Gynecologic Oncology Division, OBGYN Department, Stanford University School of Medicine, Stanford, CA 94305, USA

**Keywords:** steady-state and dynamic distribution, gynecological cancers, quantitative fluorescence microscopy

## Abstract

**Simple Summary:**

The distribution and mobility of proteins inside the living cell can be used to differentiate cancer from normal cells. This review highlights differential protein distribution of two exemplary proteins, beta-catenin and CapG, and their role in gynecologic cancers. Recognizing differential protein distribution in cancer cells may have diagnostic and therapeutic implications.

**Abstract:**

It is well-established that cancer and normal cells can be differentiated based on the altered *sequence* and *expression* of specific proteins. There are only a few examples, however, showing that cancer and normal cells can be differentiated based on the altered *distribution* of proteins within intracellular compartments. Here, we review available data on shifts in the intracellular distribution of two proteins, the membrane associated beta-catenin and the actin-binding protein CapG. Both proteins show altered distributions in cancer cells compared to normal cells. These changes are noted (i) in *steady state* and thus can be visualized by immunohistochemistry—beta-catenin shifts from the plasma membrane to the cell nucleus in cancer cells; and (ii) in the *dynamic* distribution that can only be revealed using the tools of quantitative live cell microscopy—CapG shuttles faster into the cell nucleus of cancer cells. Both proteins may play a role as prognosticators in gynecologic malignancies: beta-catenin in endometrial cancer and CapG in breast and ovarian cancer. Thus, both proteins may serve as examples of altered intracellular protein distribution in cancer and normal cells.

## 1. Introduction

Cells are individual compartments that differ in composition from the exterior. These compartments are generally maintained by lipid membrane systems that prevent homogenous mixing of interior and exterior. Furthermore, various intracellular compartments exist that may or may not be enclosed by lipid membranes. These compartments limit the exchange of molecules and permit the buildup of concentration gradients, but also employ mechanisms that allow transport across membranes and thus the exchange between compartments. Thereby, certain molecules distribute differentially between cellular compartments as well as exterior and cell interior. Examples of differential molecular distribution are symmetry-breaking and polarization events during embryonic development. Similarly, shifts in compartmentalization occur during carcinogenesis. Although less studied, these shifts in compartmentalization could be used to differentiate normal and cancer cells. 

Cancer has been recognized as a disease of the genome. Experimental emphasis has been placed on analyzing its genome, differences in genomic sequences and protein expression in normal and cancer. Rapid developments in sequencing capabilities facilitated these studies including the Human Genome Project [1] and The Cancer Genome Atlas (TCGA) [2,3]. In the following, we present two examples that illustrate differential protein distribution and compartmentalization in cancer and normal cells. One example shows differences in the *steady-state* distribution, the other one differences in the *dynamic* distribution. The former can be visualized using immunohistochemistry, while the latter can only be made visible using the tools of quantitative fluorescence live-cell microscopy. Both protein re-distribution scenarios discussed here have been described in gynecologic malignancies and may play a role as prognosticators in these cancers; beta-catenin in endometrial cancer and CapG in breast and ovarian cancer. 

## 2. Beta-Catenin

Beta-catenin is a dual function, or so-called moonlighting protein. It has two major roles in the cell: It is part of (i) the cell adhesion complex E cadherin and (ii) the canonical Wnt/beta-catenin signaling pathway. In the late 1980s, beta-catenin was discovered as a structural protein of the E-cadherin complex, a key molecule of Ca^2^-dependent cell adhesion. Hence, the name catenin (catena—Latin chain) linking E-cadherin to cytoskeletal structures [4]. 

In the early 1980s, Nusse and Varmus discovered a new mouse proto-oncogene, named Int1 (integration 1) [5]. Later it was realized that Int1 had already been characterized in *Drosophila* and was known as Wingless (Wg). Wingless is the main component of the Wnt signaling pathway. The name Wnt is a portmanteau from Wingless and Int-1. It was further recognized that the armadillo protein, a main component of the Wnt signaling pathway in *Drosophila*, is structurally and functionally homologous to the mammalian beta-catenin. 

In the canonical Wnt/beta catenin pathway, Wnt glycoproteins bind to Frizzled (Fzd) receptors and Lrp co-receptors on the plasma membrane which results in the phosphorylation and thereby inactivation of the beta-catenin destruction complex. The multi-protein beta-catenin destruction complex consists of APC (adenomatous polyposis coli), axin, CK1α (casein kinase 1 alpha), and GSK3β (glycogen synthase kinase 3 beta). Once the destruction complex has been deactivated, beta-catenin accumulates in the cytoplasm and then shuttles to the cell nucleus where it binds to the transcription factor complex Tcf/Lef (T-cell factor/lymphoid enhancement factor). Multiple nucleocytoplasmic import and export mechanisms have been described. Target genes include *Jun*, *c-Myc*, and *CyclinD-1*. If Wnt does not bind to the Frizzled receptors, the beta-catenin destruction complex is not inactivated, but instead phosphorylates beta-catenin which results in its ubiquitylation and ultimately the proteasomal degradation of beta-catenin.

### 2.1. Beta-Catenin and Cancer

Alterations on any level of the Wnt/beta-catenin signaling pathway can promote cancer development: overexpression of the Wnt ligands, alterations in the Fzd receptor or Lrp co-receptor, mutations in the destruction complex, and mutations in beta-catenin itself. A prime example for alterations in the destructions complex are APC mutations resulting in decreased beta-catenin phosphorylation. Mutations in APC can cause familial adenomatous polyposis (FAP), the main hereditary factor in colorectal cancer. It was noted that the retention and not the complete loss of the first 20 amino acid repeat of APC that can bind beta-catenin and regulate its activity to some extent is favoring colon cancer formation. The unrestricted activity of beta-catenin by the complete loss of inactivating APC, however, results in further increased signaling and risk of cell death. This was postulated as the ‘just-right’ signaling model [6]. In a ‘three-hit’ hypothesis it has been stated that in some colorectal cancers Wnt signaling is modulated by copy number changes or other ‘third-hits’ of APC [7]. The common end pathway of alterations in the canonical Wnt/beta-catenin signaling pathway is that beta-catenin accumulates in the cytoplasm and then shuttles into the cell nucleus. 

Aside from its role in colon cancer, beta catenin was described to play a role in many cancers including breast cancer and the breast cancer microenvironment [5,8,9,10], hepatocellular carcinoma [11], and pancreatic cancer [12]. 

### 2.2. Change of the Steady-State Distribution of Beta-Catenin in Endometrial Cancer

**Figure 1:**
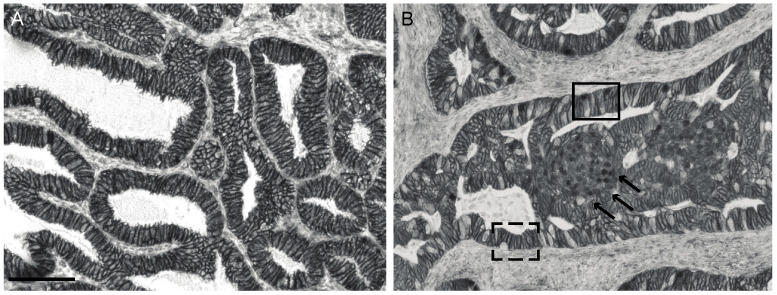
Steady-state distribution of beta-catenin in normal and cancer cell. (**A**) Membranous stain in endometrial cancer with normal distribution of beta-catenin. (**B**) Nuclear stain in endometrial cancer with aberrant beta-catenin (black box). Morule, a form of squamous metaplasia (black arrows). Normal membranous stain (black dashed box). Scale bar: 100 μm.

In 2013, The Cancer Genome Atlas (TCGA) suggested a subclassification of endometrial cancers into four groups based on their molecular profiles [13]. One of the four groups, the microsatellite-stable, low-copy number group showed *CTNNB1* mutations in the high frequency of 52%. Most mutations were found in exon 3 of the beta-catenin gene, which is thus a mutational hotspot. A mutation in this gene location results in a missense of the GSK3β consensus site, thus prevents the destruction complex from binding and stabilizes beta-catenin. This stabilization results in the re-distribution of beta-catenin from the plasma membrane to the cell nucleus. Wright et al. found this mutation in 16% of all endometrioid endometrial adenocarcinomas [14]. The redistribution of beta-catenin can be readily detected using immunohistochemistry. Costigan et al. reported a high correlation (*p* < 0.0001) of exon 3 mutations identified by sequencing with the beta-catenin redistribution into the cell nucleus detected by immunohistochemistry in endometrial cancer. Nuclear beta-catenin immunohistochemistry stain showed a 91% sensitivity and an 89% specificity. The positive and negative predictive value in this study were 86% and 93%, respectively [15]. Thus, IHC can be used as a sufficiently reliable tool to detect exon 3 beta-catenin mutations in endometrial cancer cells. Figure 1 provides an example of the steady state redistribution of beta-catenin in endometrioid endometrial cancer. Examples of membrane and nuclear beta-catenin stain are shown. 

Based on clinical data, the prognostic relevance of a beta catenin mutation and redistribution in endometrial cancer is still discussed. However, limited data suggest that a beta-catenin mutation in endometrial cancer increases the risk of recurrence in low-risk and low-stage endometrial cancers including the risk of distant recurrences [15,16]. To establish beta-catenin as negative prognosticator of low-grade, low-stage endometrioid endometrial cancer, further and larger clinical studies are needed. 

## 3. CapG 

CapG is a relatively small 39 kDa actin-binding protein of the Gelsolin family [17,18]. In contrast to other Gelsolin-related actin-binding proteins CapG lacks a nuclear export sequence. Thus, it is the only member of its family that distributes diffusively all throughout the cell, i.e., in the cytoplasm and the cell nucleus. The function of CapG in the cytoplasm has been well-established. It binds Ca^2^-dependent the rapidly growing plus ends of actin filaments, by placing a ‘cap’ on actin filaments (capping like Gelsolin). CapG does not sever [17] or nucleate new actin filaments. The capping stops the linear elongation of actin filament but promotes new filament nucleation by Arp 2/3 and results in the dense dendritic actin meshwork at the leading edge of a cell [19]. Cytoplasmic CapG has been shown in many studies to be involved in the actin-based cell motility and membrane ruffling [20,21]. The function of CapG in the cell nucleus, however, remains unknown. It has been hypothesized that the nuclear fraction of CapG and not only the cytoplasmic fraction promotes cell motility and invasiveness [22,23]. The underlying mechanisms of such hypothesized function of the nuclear CapG fraction are unclear.

### 3.1. CapG and Cancer 

In comparison with beta-catenin, few is known about the role of CapG in cancer. CapG was described within the German Human Genome project to be overexpressed in breast and ovarian cancer [24,25]. It may be particularly upregulated in breast cancer cells that form bone metastases [26,27,28]. There are reports of overexpression of CapG in pancreatic cancer with a reportedly immunohistochemistry nuclear stain that correlated with increasing tumor size [29]. In bladder cancer and its surrounding tumor microenvironment, CapG was found to be overexpressed compared to normal bladder tissue. CapG expression correlated with stage, grade, tumor size and shorter time to recurrence in bladder cancer [30]. Other studies report overexpression of CapG in ocular melanoma [31], clear cell renal cell carcinoma [32], mesothelioma [33], glioblastoma [34,35,36,37], and hepatocellular carcinoma with vascular invasion [38]. 

### 3.2. Change in the Dynamic Distribution of CapG in Breast Cancer

**Figure 2:**
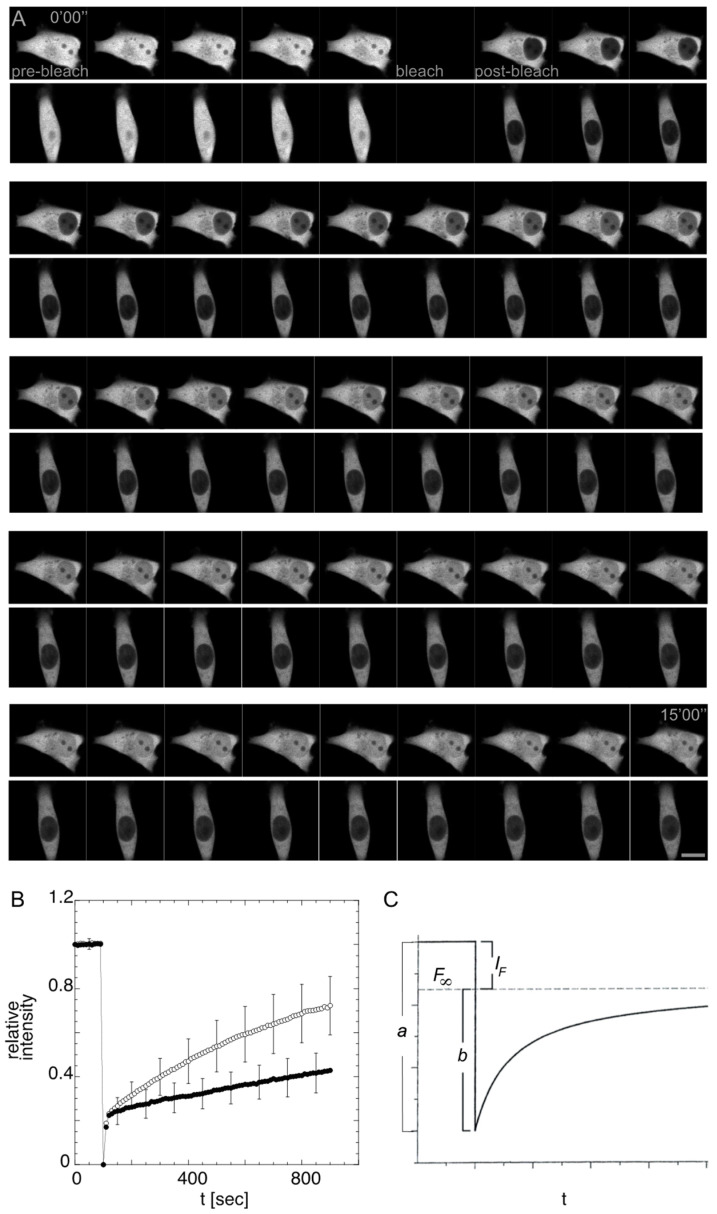
Dynamic distribution of CapG in normal and cancer cells. (**A**) Fluorescence recovery after photobleaching (FRAP) experiments show differences in dynamic nucleocytoplasmic distribution of CapG in breast cancer cell line (MDA-MB-231, upper panel) and immortalized normal breast epithelial cell line (MCF-12A, lower panel). Representative images from a time lapse, depicted only every second image from total of 90 images. Scale bar 10 μm. (**B**) Plot of mean normalized relative fluorescence intensity in the cell nucleus over time for MCF-12A (filled circles) and MDA-MB-231 (open circles). Error bars indicated standard deviations [39]. (**C**) Schematic of Fluorescence after Photobleaching Recovery curve fit to a single exponential function. *a* is the fraction of fluorescence intensity initially bleached, *b* the fraction that recovers over time, and *I_F_* the immobile fraction, i.e., the fraction of immobile irreversibly bleached molecules that is not replaced by still fluorescent molecules. τ is the characteristic recovery time, i.e., the time by which two-thirds of the initial fluorescence intensity have been regained.

As in other cells, CapG distributes diffusively in both the cell nucleus and cytoplasm of the highly invasive breast cancer cell line MDA-MB-231 and the nearly normal breast epithelial cell line MCF-12A. Transfected with CapG-GFP, there is no apparent difference in fluorescence intensity in cytoplasm and nucleus in both cell lines. However, the nucleocytoplasmic shuttling in these two cell lines was found to be different [39]. CapG-GFP shuttles faster into the cancer cell nucleus of the MDA-MB-213 cell than into the cell nucleus of MCF-12A. This difference in dynamic distribution was revealed by Fluorescence after Photobleaching (FRAP) experiments and cannot be detected by merely recording the steady-state distribution of fluorescent molecules. FRAP or fluorescence photobleaching recovery (FPR) examines the ensemble dynamics of molecules and was originally introduced in the mid-1970s to determine the dynamics of plasma membrane proteins [40,41]. The advent of the green fluorescent protein (GFP) and advances in laser scanning microscopes have allowed the technique to be more broadly applicable since the mid-1990s [42]. Using FRAP, fluorescent molecules in a cellular compartment or region of interest are irreversibly bleached with high laser intensity. Thereby, the equilibrium of fluorescent molecules is disturbed, and two distinct populations of (i) irreversibly bleached and (ii) still fluorescent molecules are created. Then, it is monitored over time with low laser intensity how the equilibrium of fluorescent molecules recovers. This fluorescence recovery reveals the underlying ensemble dynamics of a protein of interest. For both cancer and normal cells, the Cap-GFP molecules in the cell nucleus were irreversibly bleached as shown in Figure 2A. By monitoring the fluorescence increase in the cell nucleus with low laser intensity, the net transport of still fluorescent CapG-GFP molecules into the cell nucleus was visualized. Analyzing the raw data, mean grey intensities of the regions of interest Roi(t) were background BG(t) subtracted. To correct for loss of fluorescence intensity by the bleaching event and acquisition photobleaching as well as to correct for laser fluctuations, the fluorescence intensity ratio of fluorescence in the cell nucleus Roi(t) relative to the cytoplasm Tot(t) was assessed. This ratio was normalized to the initial fluorescence intensity ratio in cytoplasm and cell nucleus as provided below [43].

$$F\left(t\right)=\frac{Roi\left(t\right)-BG\left(t\right)}{Tot\left(t\right)-BG\left(t\right)}\times \frac{Tot\left(t_{0}\right)-BG\left(t_{0}\right)}{Roi\left(t_{0}\right)-BG\left(t_{0}\right)}$$

The experimentally determined fluorescence intensity over time F(t) (Figure 2B) can be fit to a single-exponential function as below [42] or, likely more precisely, to a Bessel function [44].

$$F\left(t\right)=1-\left(a-b\left(1-e^{-\lambda t}\;\right)\right)$$

In a single-exponential function, *a* is the fraction of fluorescence intensity initially bleached, *b* the fraction that recovers over time, and $I_{F}$ the immobile fraction that does not recover on the timescale of the experiment, i.e., the fraction of immobile irreversibly bleached molecules that is not replaced by still fluorescent molecules (Figure 2C). The reciprocal of λ is the characteristic recovery time τ, i.e., the time by which two-thirds of the initial fluorescence intensity have been regained. The slope of the curve gives the characteristic recovery time τ. Both parameters, τ and $I_{F}$, distinguish the CapG-GFP transport kinetics in MDA-MB-231 as compared to MCF-12A as shown in Table 1 [39]. 

In fact, τ and $I_{F}$, are independently distinguishing parameters of cancer and normal cells. $I_{F}$ remains different, even if the recovery times vary as shown in the analysis of different subgroups [39]. The prognostic and clinical relevance of these proof-of-principle findings is at the time uncertain and will require further studies. The described difference of nucleocytoplasmic CapG shuttling has not been tested in clinical samples.

## 4. Discussion

**Figure 3:**
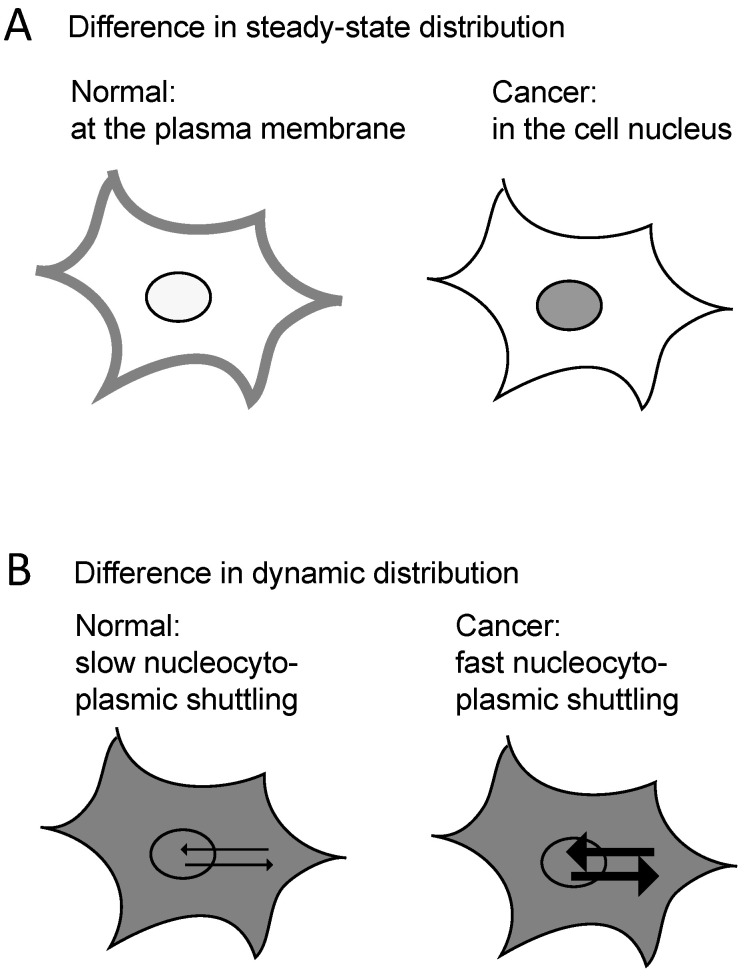
Schematic of intracellular compartment shifts. Scenario (**A**) Difference in steady-state distribution, e.g., beta-catenin. Scenario (**B**) Differences in dynamic distribution, e.g., CapG.

Beta-catenin in endometrial cancer and CapG in breast cancer are examples of differential protein distributions in normal and cancer cells. Beta-catenin shows a difference in its steady-state distribution and can be analyzed using immunohistochemistry. CapG shows a difference in its dynamic distribution between the cytoplasm and the cell nucleus that can only be revealed by quantitative fluorescence live-cell microscopy (Figure 3). To our knowledge, the CapG distribution is the only dynamic protein distribution described thus far that differentiates normal and cancer cells using FRAP analysis. The starting point for both studies on different protein distributions was the analysis of the genome which showed differences in sequence and protein expression, respectively. Beta-catenin and CapG may be examples for what further analyses of the molecular distribution and compartmentalization may reveal aside from analyses of the genome. 

Preventing shifts in compartmentalization could be used therapeutically. Currently, there are ongoing clinical trials exploring the interference with intracellular shuttling and in particular nucleocytoplasmic shuttling. Selinexor, a synthetic small molecule, blocks the nuclear export protein exportin 1 (XPO1) or also so-called chromosomal maintenance region (CRM1) by interfering with the Ran GTP-GDP cycle. It is not fully understood how far this interference results in anti-cancer effects. It has been hypothesized that XPO1 exports particularly tumor suppressor proteins from the cell nucleus so that they cannot exert their tumor suppressive effects. It is unclear if there are other consequences of the disruption of this highly conserved export mechanism on the cellular level. In initial clinical trials Selinexor was used for various cancers including multiple myeloma and lymphoma. A phase II trial studied Selinexor in heavily pretreated gynecological cancers [45]. The SIENDO trial is a phase III trial in recurrent or advanced endometrial cancer [46] and has recently been closed for recruitment. Data were presented at this year’s meeting of the European Society of Medical Oncology (ESMO) and showed improvement in recurrence-free survival with Selinexor maintenance treatment following chemotherapy [47]. These clinical data may underline the importance of analyzing intracellular protein distribution and compartmentalization in normal and cancer.

The biophysical techniques of quantitative fluorescence microscopy may help identify and analyze differences in molecular distributions. These techniques are especially well suited for the analysis of dynamic molecular distributions that are not accessible to classic immunohistochemistry. In this respect, quantitative fluorescence microscopy may contribute to personalized medicine and its current analyses of genome sequence and protein expression levels.

## 5. Conclusions

Beta-catenin shifts its steady-state distribution in endometrial cancer cells from the plasma membrane to the cell nucleus which can be shown using immunohistochemistry. CapG exhibits faster nucleocytoplasmic shuttling in breast cancer cells than in normal breast epithelial cells as shown in a proof-of-principal study using quantitative live-cell microscopy. Intracellular protein compartmentalization and mobility can be used to differentiate normal from cancer cells and may have diagnostic, prognostic, and therapeutic implications. 

## Figures and Tables

**Table 1:** The recovery time τ and the immobilized fraction $I_{F}$ are different in normal (MCF-12A) and cancer (MDA-MB-231) cells.

	Normal	Cancer
τ	1736 ± 193 s	730 ± 11 s
$I_{F}$	30.8 ± 6%	3.3 ± 1%
